# Computerized Motion Sensitivity Screening Tests

**DOI:** 10.4103/0974-9233.80714

**Published:** 2011

**Authors:** Viroj Wiwanitkit

**Affiliations:** Wiwanitkit House, Bangkhae, Bangkok Thailand 10160

Sir,

I read the recent publication on “computerized motion sensitivity screening tests” with great interest. Babalola *et al*. concluded that “the MSST (motion sensitivity screening tests) appears to be a useful test in community-wide screening and diagnosis as it reflects the general level of ocular pathology and specifically, optic nerve disease.”^1^ However, more information is required to support the clinical utility of this paper. For example, data on the sensitivity, specificity, and accuracy of the test are needed in order for the reader to understand the reliability of the test. In addition, the cost and cost effectiveness may be helpful in appraisal of incorporating this test for the interested reader. Finally, one questions the suitability of the test as the percent of subjects who completed the test was not high.
